# Risky Sexual Practice among Street Dwelling People in Southern Ethiopia: A mixed-Method Study

**DOI:** 10.4314/ejhs.v31i3.4

**Published:** 2021-05

**Authors:** Negash Wakgari, Terefe Woyo, Emnet Kebede, Hirut Gemeda, Samson Gebremedhin, Wakgari Binu

**Affiliations:** 1 Department of Midwifery, Ambo University, Ambo, Ethiopia; 2 Department of Midwifery, Hawassa University, Hawassa, Ethiopia; 3 School of Public Health, Addis Ababa University, Addis Ababa, Ethiopia; 4 School of Public Health, Wolaita Soddo University, Wolaita, Ethiopia

**Keywords:** Risky sexual practice, Street Dwellers, Southern Ethiopia

## Abstract

**Background:**

The number of street dwellers in major cities in Ethiopia is rapidly increasing. However, their sexual health needs are not that much studied. Hence, this study assessed risky sexual practice and associated factors among street dwelling people in southern Ethiopia.

**Methods:**

A cross-sectional study employing a mixed method was conducted. For the quantitative part, a snowball sampling technique was made to conduct face-to-face interviews among 842 respondents. In-depth interviews among street dwellers and key informant interviews among stakeholders were conducted to collect qualitative data.

A pre-tested and structured interviewer-administered questionnaire was used to collect data. The collected data were entered using Epidata and exported to SPSS for analysis, and qualitative data analyzed by thematic analysis approach.

**Results:**

About one third, 266(31.6%), of the participants had risky sexual practices within the last year of the study period. Sexual violence such as gang rape and same-sex practice were reported qualitatively. Male respondents (AOR: 3.24, 95%CI: 2.09–5.02) had a more likelihood of risky sexual practice than females. Living in Dilla (AOR: 9.62, 95%CI: 4.49–20.58) and Wolaita Soddo towns (AOR: 14.35, 95%CI: 6.29–32.69) had also a more likelihood of risky sexual practice than living in Hawassa. Moreover, the daily average income of 21–50 Birr (AOR: 0.52, 95%CI: 0.29–0.92) had a less likelihood of risky sexual practice compared to those with a daily average income of 5–20 Birr.

**Conclusion:**

Risky sexual practice among street dwelling people is found high. The Federal Ministry of Health and other stakeholders should work to cut risky sexual practices among street dwelling people.

## Introduction

A person's sexuality is shaped by his/her values, attitudes, behaviors, and all the ways in which he/she has been socialized (1). Sexuality and disclosure of information related to sexual matters is considered to be a social taboo in certain society (2). Many young people engage in sexual activity before marriage and do so at an early age often without any protection against pregnancy and sexually transmitted disease (STDs) (3). This could increase the sexual and reproductive health risk of street dwelling people in the general and female street dwellers in particular (2). Street dwellers are people literally living on the streets abandoned by their families, or they may have no family members left alive and move from friend to friend, or live in shelters (4,5). In general, street dwellers are at greater risk of sexual and reproductive health problems due to risky sexual behaviors (2,6).

Sexual violence including rape is very common among street dwelling people (7,6). Moreover, most females living on the street are sexually active and vulnerable to sexual abuse and exploitation compared to street males, while commercial sex work and rape are prevalent among both sexes (2). Similarly, most of the girls living on the street feel sexual abuse as unavoidable violence for girls particularly for newcomers and young dwellers. Hence, they admitted being involved in sexual activity of commercial nature as a means of survival (8,9).

Reproductive health service use by young people is limited in Ethiopia due to the lack of adequate facility, health worker attitudes, cultural barriers, and financial vulnerability of the adolescents (10–14). Further, youth-friendly service utilization by street dwelling people is low because they have not been targeted and could not easily access reproductive health services (15).

The number of street people in major cities of Ethiopia is dramatically increasing from time to time because of financial problems and due to their prior life situation (16,2). In addition, circumstances in which they live and work increase their vulnerability to sexual exploitation and abuse and put them at a higher risk of unwanted pregnancies, STD, and mental health problems (7,17–19). The problem was further compounded by the lack of access to sexual and reproductive health information and services (19,20).

Risky sexual practices from the reproductive health perspective are of concern for which scientific investigation is lacking among both sexes (21,12). Even though studies on the sexual behavior of street youths in Ethiopia indicated that they are sexually active at an early age and practice risky sexual behavior (4,20,22) one important limitation of these studies was that they were only conducted among children and also addressed only female street dwellers (20,15,18,16,4,21). Therefore, this study aimed to assess risky sexual practice and its associated factors among street dwelling reproductive age groups of people in southern Ethiopia.

## Materials and Methods

**Study design and setting**: A mixed-method study design was conducted among street dwelling people in Southern Nation, Nationality, and People Regional State (SNNPRS) from February 12 to March 10, 2019. SNNPRS is one of the nine Federal Democratic Republic of Ethiopian regional states and the third-largest region in the country comprising an estimated 18, 416,289 inhabitants. Hawassa is the capital city of the state with an estimated number of 328, 875 inhabitants and a rapidly growing number of street children and women (20). All street dwelling reproductive age groups in the SNNPRS were considered as the study population (23).

**Sample size and sampling procedure**: The sample size was determined by using the single population proportion formula with the following assumptions: 95% confidence level, 5% margin of error, and taking 50% proportions (p) since there are no earlier studies conducted on similar study subjects addressed both sexes. A simple random sampling technique was employed to select seven cities (Arba Minch, Dilla, Durame, Hawassa, Mizan Aman, Soddo, and Yirgalem) among fifteen cities in the region. A preliminary survey was conducted to determine an estimated number of street reproductive age group and the venues that street dwellers often used for the purpose of survival work. Finally, 845 samples were allocated proportionally to each selected city. Then, snowball sampling technique was used to recruit the representative sample size. A data saturation point was used to determine the sample size for the qualitative study. Accordingly, a total of twenty-one in-depth interviews and ten key informant interviews were carried out (23).

**Data collection tools and procedures**: The first set of data collection tools constitutes a pre-tested and structured intervieweradministered questionnaire to collect quantitative data. Different relevant literatures were reviewed to develop a tool that addresses the objectives of the study (3,8–15,18–20,21). The pre-test was conducted among 50 street reproductive age groups in Hosanna Town. The result of the pre-test was used to modify the instrument. The questionnaire was designed to assess socio-demographic characteristics, sexual activity, and education. Four Bachelor of Science in midwifery who had experience in quantitative and qualitative field data collection were used as data collectors, and two Master's of Science degree holders were employed as a supervisors (23). The second set of data collection tool constitutes unstructured questions which were designed to serve as a guide for in-depth and key informants' interviews to collect qualitative data.

A separate guiding checklist was used for male street respondents with a history of risky sexual practice and female street respondents with a history of sexual assault. The guiding checklist for an in-depth interview constitutes sexual experience (when and how sexual act occurred). To dig out all basic information, more neutral questions were asked first, and then the most important and more sensitive questions were asked at last for both qualitative and quantitative data collections from the street dwellers.

Similarly, a guiding checklist for key informants' interview constitutes service year, service they had been providing for street people, reproductive health problems that street people had faced and responsibility of their sectors to solve the reproductive health problems of street people and how they had approached street people in their cities. Based on their permission, the voices of the respondents were recorded by the sound recorder, and notes were taken during the interview for the qualitative part of the study.

**Data quality control**: The quality of data was assured by properly designing and pre-testing the tool, and giving training for the data collectors and supervisors before the actual data collection. The data collectors and supervisors were trained before the actual work about the aim of the study, procedures, how to approach the study participants, and data collection techniques. Every day after data collection, data were reviewed and checked for completeness, accuracy, and clarity by the supervisors, and the necessary feedback was offered to data collectors the next morning. Data cleanup and cross-checking were done before analysis (23).

**Data management and analysis**: The quantitative data were checked manually for completeness, coded, and entered into Epidata version 3.1. After the entry, the data was exported to SPSS version 23.0 for further analysis. The descriptive results were presented in the form of tables, figures, and texts using frequencies and summary statistics such as mean, standard deviation, and percentage. Binary logistic regression was used to determine the association of independent variables with the risky sexual practice and the association of all independent variables was also checked with the response variable at the same time to adjust the influence of the likelihood of various independent variables (confounding effects) on the outcome variable. All variables having a pvalue less than 0.25 were included in multivariate analysis. Odds ratio with 95% confidence interval and p-value was used to identify the significant variables. And, variables with P-value less than or equal to 0.05 were considered significant.

Regarding the qualitative part, responses from both in-depth and key informant interviews were transcribed in their respective local languages and then translated into English. Keywords and sentences were extracted from the transcribed interview for textual analysis (23).

**Operational definitions**: Individuals who are dependent on the street for their life and sleep on the street were considered as street “off” while people who depend on the street for their subsistence, but usually return home at night were considered as street “on” (5,23).

**Risky sexual practice**: Street reproductive age groups who had sex with a non-regular sexual partner or exchanged sex for money, or have more than one sexual partner, or have had rape in the last one year of the study, or not use condoms or use inconsistently in the last three months. Those respondents who had one or more of these were considered as having risky sexual practice.

**Ethics**: Ethical approval was obtained from Hawassa University, College of Medicine and Health Sciences Ethical Review Board with a permit number of IRB/015/10. A letter of permission was obtained from the SNNPR Health Bureau to respective zonal health departments. Each respondent was informed about the objective of the study that it contributes necessary information for policymakers and other concerned bodies. They were also informed that all the data obtained from them would be kept confidential by using codes instead of any personal identifiers and is meant only for the purpose of the study. Finally, written and verbal consent was obtained from each study participant prior to the data collection (23).

## Results

**Socio-demographic characteristics of the respondents**: A total of 842 street dwellers were included in the study with a response rate of 99. 6%. More than half, 503(59.7%), of them were males. The mean age of the respondents was 22.95 years (SD: ±6.79) (Table 1).

**Table 1:** Socio-demographic characteristics of street dwellers in Southern Ethiopia, 2019 (n=842)

Variables	Frequency	Percent
**Sex**		
Male	503	59.7
Female	339	40.3
**Age in years**		
1518 years	246	29.3
19-25	407	48.3
≥26 years	189	22.4
**Marital status**		
Single/not ever married	521	61.9
Married	233	27.7
Divorced/separated	73	8.7
Widowed	15	1.8
**Educational status**		
Illiterate	187	22.2
Elementary	542	64.4
High school	102	12.1
College and above	11	1.3
**Schooling status**		
In school	97	11.5
Out of school	745	88.5
**With whom currently living**		
Alone	257	30.5
Peers	382	45.4
Boyfriend/Girlfriend	135	16.0
Parents	50	5.9
Relatives	18	2.1
**Place of current residence**		
Hawassa	281	33.4
Soddo	165	19.6
Dilla	138	16.4
Arba Minch	117	13.9
Mizan Aman	108	12.8
Yergalem	20	2.4
Durame	13	1.5
**Place of residence prior to starting street life**		
Major urban town	313	37.2
Small town	369	43.8
Rural area	160	19.0
**Type of Street**		
Street “on”	274	32.5
Street “off”	568	67.5
**Duration of stay on the street**		
< 5 years	621	73.8
5–10 years	168	20.0
>10 years	36	4.3
Could not estimate	17	2.0
**Average daily income in Birr**		
5–20	136	16.2
21–50	518	61.5
≥51	654	77.7

**Reasons for joining street life and source of income**: The reasons for joining street life reported by street dwelling people were: looking for job 351(41.7%);family conflict 167(19.8%), peer influence 135(16.0%);poor family 85(10.1%); family dissolution 64(7.6%), and family forced one to leave home 40(4.7%). Furthermore, the major source of income in the last two weeks was a daily labour in 264(31.4%), begging in 135(16.0%), survival sex work in 161(19.1%), and any occasional work in 433(51.4%).

**Attitude towards street dwellers**: Regarding the attitude of the communities towards street dwelling people, lack of awareness by the community about street dwelling people and assuming all street dwellers as a burglar by the community members were mentioned during an interview. Moreover, the attitude of the government sectors particularly police officers found to be an additional challenge for street-dwelling people. The communities and family have fear on the street dwellers because they think that they might negatively influence the behavior of their child and even some families challenge one to receive their child back to home from street life. The key informant interview from Integrated Service on Health and Development Organization coordinator also indicated that: “…*if the street dwellers report to police that he/she is raped, usually they claimed them as they provoke for the sexual act, this paramount if she/he is a commercial sex worker*.”

In supporting the above idea, a senior staff from Justice and Security Office said: “*Some of our communities think that all street dwelling people are bad-mannered and aimless. Even if they want to be engaged in daily labor, most of our community could not believe street dwelling people. This is very dangerous*.”

**Challenges and suggested solution**: Participants of the study suggested different solutions to solve the sexual and reproductive health problem of street dwelling people. Some of the challenges reported by respondents were: poor integration between different government sectors to solve the reproductive health problem of street dwelling people and the rapid rise of the number of street dwelling people in major towns of Ethiopia. A senior staff of the Social and Labor Affairs office said: “….. *the solution is employing them immediately after the life skill training which is difficult in our case. For instance, a month before, a number of street dwellers took different life skill trainings in Addis Ababa and came back to Dilla. Then, due to lack of access to work and poor integration between different sectors, they returned to street life*.”

Moreover, a BSc nurse working at the Center of Concern shared her experience about the life of street dwelling people and suggested solutions as follows: “…..*for me, poverty reduction in a rural area and parenting skill are the most important issue to be addressed*.”

**Sexual activity and risky sexual practice**: About two-thirds, 571(67.8%), of the respondents ever had sexual intercourse. The minimum age at which they had their first sexual intercourse was 8 years with the mean age of 16. 71 years. About 56(6.8%) of the respondents thought they ever had more than ten sexual partners in their lifetime. Moreover, 266(31.6%) of the street dwellers had at least one risky sexual practice in the last year of the study period [95% CI: 28.6–34.8]. The major reasons for having sexual intercourse in the last year were exchanged sex for money 125(14.8%) and the influence of khat/alcohol accounting for 122(14.5%) (Table 2). This is also supported by the qualitative study. A 17 years old street dwelling female said the following points: *“…..I faced sexual violence when I was 10 years old......we have been facing robbery, sexual violence, and its consequences. Since I am with my husband, I am protected. It is good to have a boyfriend here on the street; that is a protective factor*.”

**Table 2:** Sexual activity and risky sexual practice of street dwellers, Southern Ethiopia, 2019 (n=571)

Variables	Frequency	Percent
**Ever had sexual intercourse**		
Yes	571	67.8
No	271	32.2
**Age at first sexual intercourse**		
8–14 years	102	12.1
15–18years	308	36.6
19 years and above	109	12.9
Don't remember	52	9.1
**Number of sexual partners ever had**		
1	162	19.2
2 and more	199	23.6
Don't remember	210	36.8
**Have you had sexual intercourse in the last year**		
Yes	334	58.5
No	237	41.5
**Reasons for having sexual intercourse in the last year**		
Exchanging sex for money	125	14.8
Rape	88	10.5
Fall in love	71	8.4
Influence of chat/alcohol	122	14.5
Marriage	35	4.2
Peer pressure	125	14.8
Personal desire	18	2.1
**Had sexual intercourse within the last 3 months**		
Yes	334	58.5
No	237	41.5
**With whom you had sex in the last 3 months**		
With my friend	141	16.8
With my husband/wife	170	20.2
I don't know with whom I had	227	26.5

**Same sexual practice**: Among the total respondents, 177(21.0%) reported that they had sexual intercourse with commercial sex workers. Similarly, 41(4.9%) of them ever had sex with a person of the same sex. Moreover, 74(8.8%) of the street dwellers reported that they had encountered rape in the last year of the study. Among these, 9(1.1%) of them had sexual intercourse with the same sex in the last year of the study. This is also supported by in-depth and key informant interview participants. A 19 years old-young street dwelling men said: *“......I was raped by my friend when I was 15 years old on the street. He raped me, and I kept silent because I was a kid while he was muscular. After that, I fear all my friends, and I take time to approach them…...”*

**Condom practice among street dwellers**: From the respondents who ever had sexual intercourse, 334(58.5%) had sexual intercourse in the last three months of the study. Among them, 309(36.7%) used condoms. The major reasons for using condom listed by respondents were: to prevent pregnancy, 113(13.4%), to protect oneself from HIV, 269(31.9%), to protect oneself from other STD, 132(15.7%), and parental insistence, 27(3.2%). The major sources of condom for respondents were 140(16.6%) “*Suk Be Derat*” which means shop at the chest, 91(10.9%) from friends and 77(9.1%) from government health institutions (Table 3). Respondents of in-depth and key informant interviews indicated that lack of knowledge about STDs and condom use were an extra problem to use condoms. Correspondingly, a 35 years-old street dwelling woman said: “*We are being exposed to reproductive health problems due to lack of knowledge about STDs, and condom use ……*.” Moreover, a 20 years old young street dwelling male also added the following points: *“…. the consequences of sexual intercourse I fear are pregnancy only. It is must use condoms if she is HIV positive; unless there is no need to use condoms*…..”

**Table 3:** Condom utilization among reproductive age street dwellers in Southern Ethiopia, 2019

Variables	Frequency	Percent
**Patterns of condom use in the last 3 months (n=334)**		
Always	157	18.6
Often	35	4.2
Irregularly	117	13.9
**Reasons for not using condom always**		
Condom decrease sexual pleasure	105	12.5
Lack of access to condom	24	2.9
Because I have married	11	1.3
My partner did not want to use a condom	12	1.4

**Factors associated with risky sexual practice**: In bivariate analysis, the factors found to be significantly associated with the risky sexual practice were: sex, age, marital status, educational status, daily average income, with whom one is living, place of current residence, place of residence before joining street life, attitude towards STDs, and any health education attended in relation to family planning. From variables found to be significant in bivariate analysis, sex, place of current residence, and daily income was found to be significantly associated with risky sexual practice in multivariate logistic regression analysis. Accordingly, being a male was 3 times more likelihood of risky sexual practice than those who were females (AOR: 3.24, 95%CI: 2.09–5.02). In addition, living in Dilla and Wolaita Soddo were about 10 and 14 times more likely to practice risky sexual behavior than those who were living in Hawassa (AOR: 9.62, 95%CI: 4.49–20.58), (AOR: 14.35, 95%CI: 6.29–32.69) respectively. Furthermore, those street dwellers with a daily average income of 21–50 Birr were about 48% less likely to practice risky sexual behavior compared to a daily average income of 5–20 Birr (AOR: 0.52, 95%CI: 0.29–0.92) (Table 4).

**Table 4:** Bivariate and multivariate analyses of factors associated with risky sexual practice among street dwellers in Southern Ethiopia, 2019 (n=842)

Variables	Risky sexual practice		(95% CI)	
	Yes	No	COR(95% CI)	AOR(95% CI)
**Sex**				
Male	115	388	2.71(2.01, 3.65)	**3.24(2.09,5.02)** **
Female	151	188	1	
**Age in Years**				
15–18	59	187	1	
19–25	151	256	0.53(0.37,0.76)	*
26 and more	56	133	0.75(0.49,1.15)	
**Educational status**				
Illiterate	23	164	1	
Elementary	190	352	0.26(0.16,0.42)	
High school	51	51	0.14(0.08,0.25)	
College and above	2	9	0.63(0.13,3.10)	
**With whom currently living**				
Alone	108	149	1	
Peers [boy/girlfriend]	138	379	1.99(1.45,2.73)	*
Parents or relatives	20	48	1.74(0.98,3.09)	
**Place of current residence**				
Hawassa	137	144	1	
Dilla	10	128	12.18(6.14, 24.15)	**9.62(4.49,20.58)** **
Wolaita Soddo	8	157	8.67(8.84 39.44)	**14.35(6.29, 32.69)** **
Durame	1	12	11.42(1.46, 88.98)	3.64(0.37,35.06)
Mizan Aman	36	72	1.90(1.19,3.02)	1.43(0.83,2.47)
Arba Minch	72	45	0.59(0.38,0.92)	0.92(0.51,1.66)
Yirgalem	2	18	8.56(1.95,37.59)	6.18(1.17,32.67)
**Place of residence prior to joining**				
**Street Life**				
Major urban town	122	191	0.29(0.18,0.47)	*
Small town	119	250	0.39(0.24,0.63)	
Rural area	25	135	1	
**Daily average Income**				
5–20 birr	31	105	1	
21–50 birr	158	360	0.67(0.43,1.05)	**0.52(0.29,0.92)** ***
51 and more	77	111	0 .43(0.26,0.69)	0.53(0.28,1.01)
**Attitude towards STDs**				
Favourable	249	462	0.27(0.15,0.45)	*
Unfavourable	17	114	1	
**Any health education attended about** **family planning in the last one year**				
Yes	92	96	2.64(1.89,3.69)	*
No	174	480	1	

* Not significant in backward stepwise logistic regression

** Significant in backward stepwise logistic regression (p<0.001)

*** Significant in backward stepwise logistic regression (p<0.05)

## Discussion

In this study, about one-third, 266(31.6%), of the street dwellers had at least one risky sexual practice in the last year of the study period [95%CI: 28.6–34.8]. This finding is similar to the study conducted in the Northwest part of Ethiopia (24). Moreover, it is higher than the study which was conducted in Bahir Dar, Ethiopia (11.4%) (8). The possible explanation for this difference is the risky sexual practice in the present study was measured with different variables such as having sex with a non-regular sexual partner, exchange sex for money, having more than one sexual partner, having rape, and not used condoms or use inconsistently, but in the study of Bahir Dar, only rape was used to measure risky sexual practice. However, the present finding is lower than the study that was conducted in Kathmandu Valley (43%) (25). This discrepancy might be due to differences in place of the study and socio-demographic characteristics of the respondents. For instance, the participants of the Kathmandu Valley study were teenagers [13–19 years] which might increase risky sexual behavior compared with the present study in which the reproductive age group [15–49 years] were involved. The other possible explanation for this difference could be the sample size and sampling procedure. In the study of Kathmandu Valley, only 90 teenagers were involved which might have an impact on the proportion of risky sexual behavior.

Furthermore, sexual violence such as gang rape following substance use and same-sex practices was reported in the qualitative study. This might be due to their living condition that lacks protection that could expose them to sexual assaults. The other possible explanation might be lack of awareness about the health consequences of sexual violence.

Males were about 3 times at more likelihood of risky sexual practice than those who were females. This might be due to the fact that male respondents had a more likelihood of multiple sexual partners than female respondents (26). Moreover, the place of current residence was also significantly associated with risky sexual practice. Living in Dilla and Wolaita Soddo towns had a more likelihood of risky sexual practice than living in Hawassa. This might be due to the difference in the accessibility of health services. Since Hawassa is the capital city of the regional state, different governmental and non-governmental organizations might be found there working on the street dwelling people that consequently improve their knowledge about sexual health. Furthermore, the daily average income of the street dwellers was also another variable influencing risky sexual practice. Daily income is crucial for the life of street dwellers that could help them to avoid sex for money. In conclusion, in this study, risky sexual practice among street dwellers is found high. Moreover, sexual violence such as gang rape following substance use and same-sex practices is reported in the qualitative study. Concerning this, sex, place of current residence, and the daily average income of the street dwelling people were significantly associated with risky sexual practice. The Federal Ministry of Health and other stakeholders should work to cut risky sexual practices among street dwelling people.
